# Explainable prediction of daily hospitalizations for cerebrovascular disease using stacked ensemble learning

**DOI:** 10.1186/s12911-023-02159-7

**Published:** 2023-04-06

**Authors:** Xiaoya Lu, Hang Qiu

**Affiliations:** 1 School of Computer Science and Engineering, University of Electronic Science and Technology of China, No.2006, Xiyuan Ave, West Hi-Tech Zone, 611731 Chengdu, Sichuan People’s Republic of China; 2 Big Data Research Center, University of Electronic Science and Technology of China, Chengdu, China

**Keywords:** Stacking ensemble model, Environmental exposure, Hospital admissions, Cerebrovascular disease, SHAP value

## Abstract

**Background:**

With the prevalence of cerebrovascular disease (CD) and the increasing strain on healthcare resources, forecasting the healthcare demands of cerebrovascular patients has significant implications for optimizing medical resources.

**Methods:**

In this study, a stacking ensemble model comprised of four base learners (ridge regression, random forest, gradient boosting decision tree, and artificial neural network) and a meta learner (elastic net) was proposed for predicting the daily number of hospital admissions (HAs) for CD using the historical HAs data, air quality data, and meteorological data in Chengdu, China from 2015 to 2018. To solve the label imbalance problem, a re-weighting method based on label distribution smoothing was integrated into the meta learner. We trained the model using the data from 2015 to 2017 and evaluated its predictive ability using the data in 2018 based on four metrics, including mean absolute error (MAE), root mean square error (RMSE), mean absolute percentage error (MAPE), and coefficient of determination (*R*^2^). In addition, the SHapley Additive exPlanations (SHAP) framework was applied to provide explanation for the prediction of our stacking model.

**Results:**

Our proposed model outperformed all the base learners and long short-term memory (LSTM) on two datasets. Particularly, compared with the optimal results obtained by individual models, the MAE, RMSE, and MAPE of the stacking model decreased by 13.9%, 12.7%, and 5.8%, respectively, and the R^2^ improved by 6.8% on CD dataset. The model explanation demonstrated that environmental features played a role in further improving the model performance and identified that high temperature and high concentrations of gaseous air pollutants might strongly associate with an increased risk of CD.

**Conclusions:**

Our stacking model considering environmental exposure is efficient in predicting daily HAs for CD and has practical value in early warning and healthcare resource allocation.

**Supplementary Information:**

The online version contains supplementary material available at 10.1186/s12911-023-02159-7.

## Background

Cerebrovascular disease (CD) is a leading cause of death and disability worldwide. The World Health Organization has reported that more than 6 million deaths can be attributed to CD each year [1]. In China, about 13 million people suffered from stroke, a subtype of CD [2]. Although hypertension, high-fat diet, smoking, and alcohol consumption are well-known risk factors for CD, evidence from epidemiological studies indicates that short-term environmental exposure, such as air pollution and extreme weather conditions, also has an important impact on the onset of CD, resulting in an increased risk of morbidity [3–5]. Additionally, toxicological studies have also presented several credible biological mechanistic pathways for the negative health effects associated with air pollution [6–8]. For example, air pollution exposure may provoke platelet activation, leading to enhanced blood coagulation and thrombosis formation [9].

The growing morbidity and high treatment cost of CD have caused a heavy burden on the limited healthcare resources. Forecasting the daily number of hospital admissions (HAs) for CD is of practical significance to optimize medical resources and protect public health by providing an early-warning signal against the impending incidence. Common traditional regression methods for time series prediction, such as the gray model, simple exponential smoothing (SES) model, and autoregressive integrated moving average (ARIMA) model, have been widely applied in predicting healthcare service demand [10–13]. The traditional methods can be easily implemented but have difficulties dealing with multi-factor effects and non-linear mapping, therefore, these studies seldom extract features from factors other than historical demand series.

Machine learning (ML) methods can overcome these disadvantages of traditional regression models [14] and have been applied by a limited number of studies to forecast the demands for healthcare services associated with environmental exposure. For instance, Qiu et al. [15] found that the light gradient boosting machine model outperformed the other five ML models they tested in predicting the peak demand days for cardiovascular disease (CVD) admissions. The researchers also identified that meteorological conditions and air pollutants substantially contributed to prediction accuracy. Bibi et al. [16] used a backpropagation neural network model to predict emergency department visits and found that the model performance was remarkably improved after considering temperature, humidity, and air pollution. Kassomenos et al. [17] discovered that the use of Artificial Neural Network (ANN) resulted in a 15% increase in the coefficient of determination (R2) compared to the Generalized Linear Model (GLM) for forecasting HAs for CVDs.

In recent years, as an advanced part of artificial intelligence, deep learning (DL) have attracted much attention in related fields owing to their strong abilities in capturing potential complex relationships among variables [18]. Khatibi et al. [19] and Wang et al. [20] proposed novel predictive models based on convolutional neural network and long short-term memory (LSTM) to predict HAs due to mental disorders and cardiopulmonary diseases, respectively.

Despite the widespread use of ML and DL models in predicting healthcare demand, these models have their disadvantages. Lightweight ML models have limited prediction capabilities, and each of them has specific predefined structures and assumptions. No single model can always be optimal in various application scenarios. DL models generally rely on massive amounts of training data and a relatively long time window for the input sequence. In this context, the stacking ensemble technique [21–23] can provide an effective solution to strike a compromise by combining the strengths of multiple lightweight ML models to achieve superior performance using limited amounts of samples. Additionally, most existing studies treated these models as “black boxes” and rarely provided explanations for them, which might reduce their acceptance by the medical community [24]. Thus, it is essential to increase the transparency of ML models in the medical domain.

In this study, we applied stacking ensemble learning based on heterogeneous lightweight ML models to forecast medical demands caused by CD considering short-term environmental exposure and explained the predictions by the SHapley Additive exPlanations (SHAP) method. The main contributions of this study can be summarized as follows:

1. A stacking ensemble model was proposed to predict daily HAs for CD using the HAs data, air quality data, and meteorological data of the previous 6 days.
2. A re-weighting method based on label distribution smoothing was integrated into the proposed model to address the label imbalance problem that broadly existed in healthcare data.
3. A post-hoc interpretation for the prediction mechanism of our proposed model was provided from global and local perspectives, which is conducive to understanding the model and exploring the factors affecting HAs for CD.

## Methods

### Data collection and preprocessing

The daily counts of HAs due to CD and stroke from January 1, 2015 to December 31, 2018 were collected from the electronic hospitalization summary reports of all the tertiary and secondary hospitals in the urban areas of Chengdu, China (a total of 1461 observations). Patients under the age of 35 or with residential addresses outside of the urban districts of Chengdu were not included in the count of daily HAs. All the causes of HAs were coded using the International Classification of Disease, Revision 10 (ICD-10), including the HAs for CD (I60-I69) and stroke (I60-I64).

Hourly air pollutant concentrations measured at six monitoring stations in the urban areas of Chengdu were obtained from the China National Environmental Monitoring Center (http://www.cnemc.cn/). The 24 h average concentrations of the particular matter with a diameter less than 2.5 µm (PM_2.5_), the particular matter with a diameter less than 10 µm (PM_10_), the coarse particular matter with a diameter between 2.5 µm and 10 µm (PM_C_), sulfur dioxide (SO_2_), nitrogen dioxide (NO_2_), carbon monoxide (CO) and the 8 h moving average concentration of ozone (O_3_) were calculated as their daily concentrations [25]. The air quality index (AQI) was also obtained, assessed using the air pollutants mentioned above. Because the missing rate of ambient air quality data was 2.73% (40/1461), we used linear interpolation, which has been reported as an effective data filling method when the missing rate is low (e.g., < 5%) [26] to fill in missing values.

Meteorological observations, including the daily average temperature (TEM) and relative humidity (RH), were derived from the Chengdu Meteorological Monitoring Database (http://data.cma.cn/).

### Feature extraction and normalization

Based on our collected data, the HAs features, environmental features, and calendar features were extracted, as shown in Table 1.

**Table 1:** Feature descriptions

Feature Name	Feature Descriptions
**HAs Features**	
HAs Lag x	Historical HAs on day x before the day for prediction, x ∈ {1, 2, 3, …, L}
HAs Lag 1L mean	Moving average of historical HAs during the previous 1 to L days
HAs Lag 1L std	Standard deviation of historical HAs during the previous 1 to L days
**Environmental Features**	
P^a^ Lag x	Historical values of P on day x before the day for prediction, x ∈ {1, 2, 3, …, L}
P Lag 1L mean	Moving average of historical P during the previous 1 to L days
P Lag 1L std	Standard deviation of historical P during the previous 1 to L days
**Calendar Features**	
DOW	Day of the week, {Mon., Tues., …, Sun.}—> {1, 2, …, 7}
MON	Month of the year, {Jan., Feb., …, Dec.}—> {1, 2, …, 12}
SEA	Season of the year, {spring, summer, fall, winter}—> {1, 2, 3, 4}
YEAR	The year, {2015, 2016, 2017, 2018}—> {1, 2, 3, 4}
TS	Timestamp, serial number from 1 to 1461
HOL	Holiday, [0,1], 1 represented the day is a holiday, while 0 represented not
WD	Workday, [0,1], 1 represented the day is a work day, while 0 represented not
FWD	First work day, [0,1], 1 represented the day is the first workday, while 0 represented not
LWD	Last work day, [0,1], 1 represented the day is the last work day, while 0 represented not

^a^P ∈ {PM_2.5_, PM_10_, PM_C_, SO_2_, NO_2_, CO, O_3_, AQI, TEM, RH}

For the time series of HAs and environmental exposure, lag features were broadly considered in epidemiological studies and HAs predictions [27, 28]. In our study, single-day lag features, namely historical values on day x (x ∈ {1, 2, 3, …, L}) before prediction, and cumulative lag features, including the moving average and standard deviation of historical values during the previous 1 to L days were extracted. Besides, L was set to 6 to represent the short-term effect of environmental exposure as most epidemiological studies [3, 4]. In calendar features, day of the week (DOW), month (MON), season (SEA), year (YEAR), and timestamp (TS) were used to depict the trends of HAs from short to long term. Holiday (HOL), workday (WD), first work day (FWD), and last work day (LWD) were extracted to present the impact of the work-rest schedule in hospitals.

We processed DOW, MON, SEA and YEAR with One-Hot Encoding [29] and normalized features using the min–max normalization [30] as formulated in Eq. (1),

1 $${X}^{^{\prime}}=\frac{X-\mathit{min}(X)}{\mathit{max}(X)-\mathit{min}(X)}$$

### Model construction

#### Stacking ensemble method

**Fig. 1:**
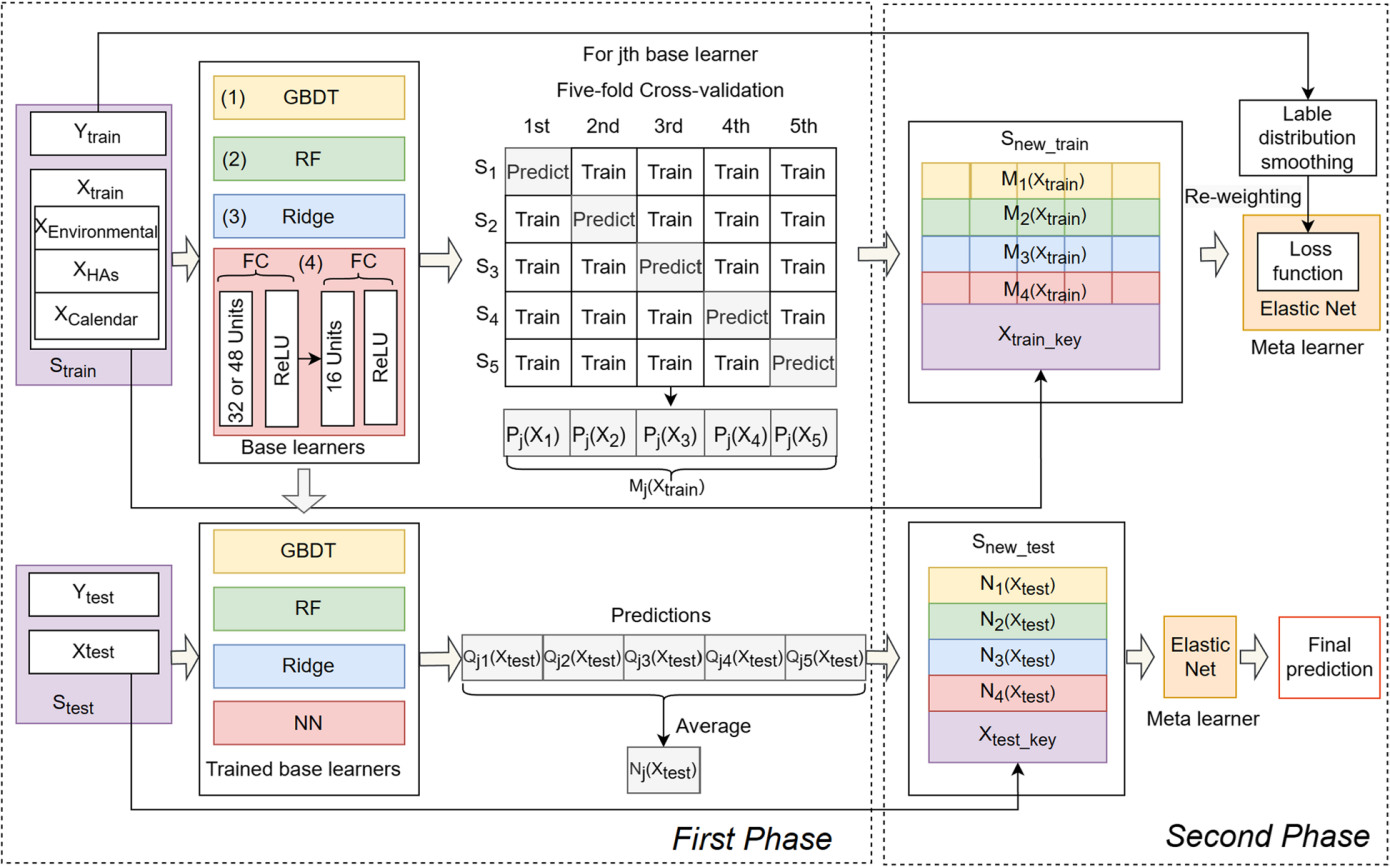
Schematic diagram of stacking model development

In this study, a stacking ensemble model comprised of four base learners and a meta learner was proposed to accurately predict the daily number of HAs for CD. As shown in Fig. 1, the development of the stacking model consists of two phases.

Initially, we split the whole dataset into a training set, which covered the period from January 1, 2015 to December 31, 2017, and a testing set, which covered the period from January 1, 2018 to December 31, 2018. We denoted them as ${\mathrm{S}}_{train} = ({\mathrm{X}}_{train}, {\mathrm{Y}}_{train})$ and ${\mathrm{S}}_{test} = ({\mathrm{X}}_{test}, {\mathrm{Y}}_{test})$, respectively, where $\mathrm{X}$ represented the feature set and Y represented the corresponding label set.

In the first phase, the four base learners, including linear regression with L2 regularization (Ridge) [31], random forest (RF) [32], gradient boosting decision tree (GBDT) [33], and ANN [34], were trained and used to make predictions as the input features for the meta learner. To avoid overfitting and improve the generalization capability, a five-fold cross-validation was implemented. We split ${\mathrm{S}}_{train}$ into five subsets in chronological order, and the *i*th subset was denoted as ${\mathrm{S}}_{i} = \left({\mathrm{X}}_{i}, {\mathrm{Y}}_{i}\right) (\mathrm{i}=1, 2, 3, 4, 5)$. At the *i*th-fold cross-validation, the *j*th base learner $(\mathrm{j}=1, 2, 3, 4)$ was trained using the subsets except S_*i*_ and made predictions on S_*i*_, which were recorded as ${\mathrm{P}}_{j}({\mathrm{X}}_{i})$. Consequently, this process was repeated a total of 20 (4 × 5) times, with each base learner making predictions once on each fold. Afterwards, the predictions generated by the *j*th base learner throughout the five-fold cross-validation were represented as ${\mathrm{M}}_{j}({\mathrm{X}}_{train}) = \{{\mathrm{P}}_{j}({\mathrm{X}}_{1}), {\mathrm{P}}_{j}({\mathrm{X}}_{2}),{\mathrm{P}}_{j}({\mathrm{X}}_{3}), {\mathrm{P}}_{j}({\mathrm{X}}_{4}), {\mathrm{P}}_{j}({\mathrm{X}}_{5})\}$ and treated as a new feature in the new training set. At the meantime, the *j*th base learner trained at the *i*th-fold cross-validation made predictions using ${\mathrm{S}}_{test}$, which were recorded as ${\mathrm{Q}}_{ji}\left({\mathrm{X}}_{test}\right)$, and the average of predictions were calculated as a new feature in the new testing set, namely ${\mathrm{N}}_{j}\left({X}_{test}\right)=\frac{1}{5}{\sum }_{i=1}^{5}{Q}_{ji}({X}_{test})$.

Furthermore, to help the meta learner decide which model to apply under a certain circumstance [23], we merged the key features selected by the base learners, namely calendar features and HAs features (as shown in Additional file 1: Fig. S1), into the new training set and the new testing set, which were denoted as ${\mathrm{X}}_{train\_key}$ and ${\mathrm{X}}_{test\_key}$, respectively. Hence, at the end of the first phase, we gained a new training set ${\mathrm{S}}_{new\_train} = ({\mathrm{X}}_{new\_train}, {\mathrm{Y}}_{train})$, where ${\mathrm{X}}_{new\_train}=({\mathrm{M}}_{1}\left({\mathrm{X}}_{train}\right), {\mathrm{M}}_{2}\left({\mathrm{X}}_{train}\right),{\mathrm{M}}_{3}\left({\mathrm{X}}_{train}\right),{\mathrm{M}}_{4}\left({\mathrm{X}}_{train}\right),{\mathrm{X}}_{train\_key})$, and a new testing set ${\mathrm{S}}_{new\_test} = ({\mathrm{X}}_{new\_test}, {\mathrm{Y}}_{test})$, where ${\mathrm{X}}_{new\_test}=({\mathrm{N}}_{1}\left({\mathrm{X}}_{test}\right), {\mathrm{N}}_{2}\left({\mathrm{X}}_{test}\right), {\mathrm{N}}_{3}\left({\mathrm{X}}_{test}\right), {\mathrm{N}}_{4}\left({\mathrm{X}}_{test}\right), {\mathrm{X}}_{test\_key})$.

In the second phase, the meta learner, i.e., the elastic net [35], was trained on ${\mathrm{S}}_{new\_train}$ and then used to make the final predictions on ${\mathrm{S}}_{new\_test}$.

For a suitable architecture of the stacking model, we have tested eight widely utilized lightweight ML models in the preliminary experiment (see Additional file 1: Table S1), and Ridge, RF, GBDT, and ANN were picked as base learners for two reasons: 1) Each of them outperformed other models in daily HAs prediction. 2) Ridge and ANN are classical linear and network ML models, respectively. RF and GBDT are ensemble tree models based on the bagging and boosting methods, respectively. Because the theories of these models are highly heterogeneous, they can obtain insight into the training data from different perspectives and eventually increase the accuracy and robustness of the stacking model [36]. Linear regression with a combination of L1 and L2 regularization (elastic net) was selected as the meta learner because it was widely used in a similar context and can prevent overfitting to a large extent [21].

#### Re-weighting with Label distribution smoothing (LDS)

In our study, daily HAs data exhibit an imbalanced distribution, where certain target values, especially peaks and troughs, have strikingly fewer observations. For classification tasks, re-sampling and re-weighting are the two main methods to address data imbalance. However, methods based on re-sampling, such as SMOTE [37] and SMOGN [38], are not directly applicable to our task, because the distance between labels was not considered and the intrinsic seasonal pattern of HAs might be damaged. We adopt the LDS method [39] to extend re-weighting schemes to regression tasks, which includes the following steps: First, discretize the continuous target space into finite bins, which can be considered as the empirical label density distribution. Then convolve the empirical label density with a symmetric kernel to calculate the effective label density that accounts for the overlap in the information of nearby labels so that the cost-sensitive re-weighting method can be utilized based on the effective label distribution.

**Fig. 2:**
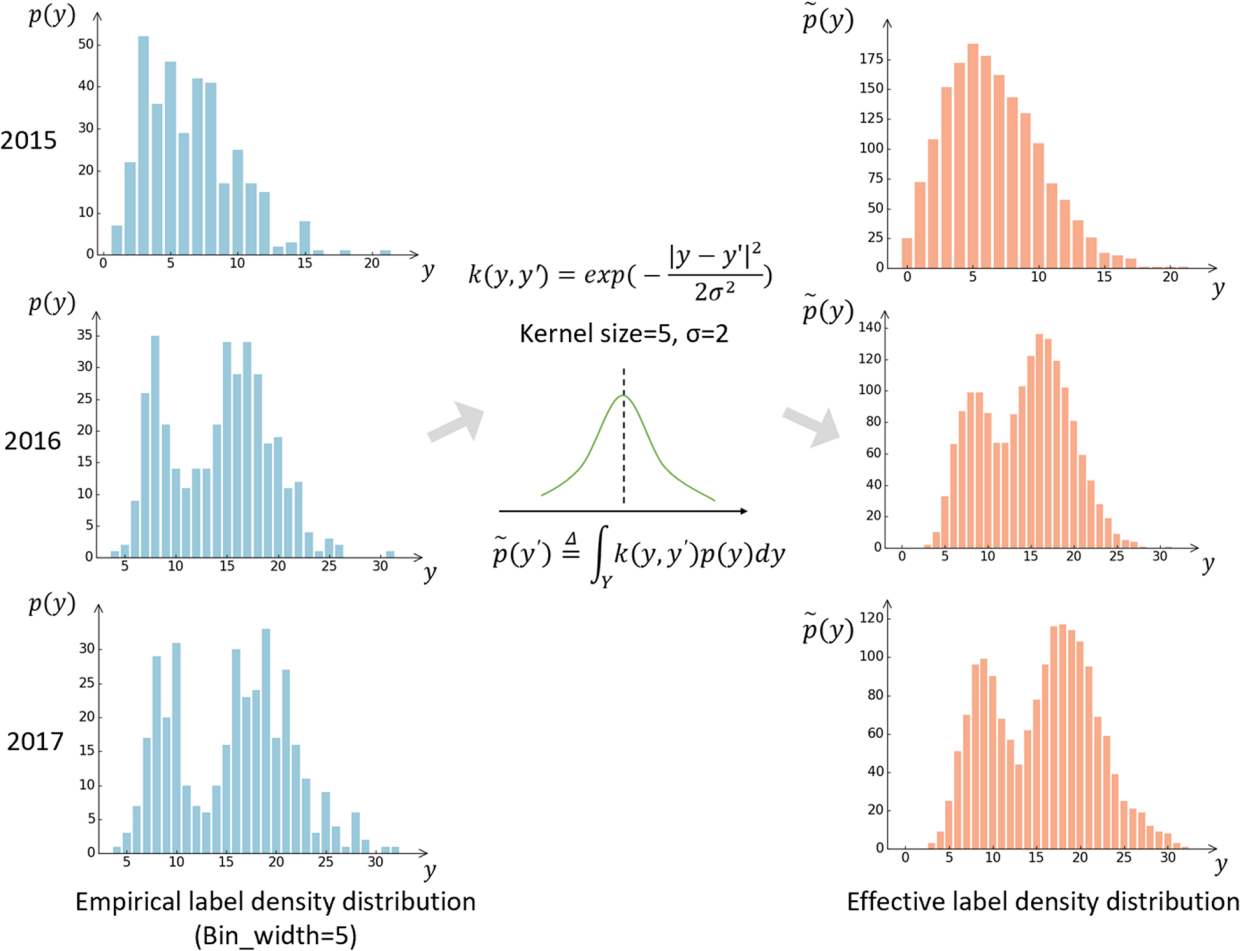
Schematic diagram of label distribution smoothing

To integrate this approach into our proposed model, in the training process of the meta learner, we used the inverse of effective label density as the weight of training samples when calculating the loss function, and given that the HAs data show a yearly rising trend and an annual seasonal pattern, it is more reasonable to calculate the LDS estimated label density within each year as shown in Fig. 2.

#### Training details and parameters

In our experiment, the ANN were completed using Keras with Tensorflow 2.4.1 as the backend. Other base learners were implemented based on the Scikit-learn 0.24.2 Python library. The computation was performed using AMD Ryzen 74800U with Radeon Graphics 1.80 GHz. In the stacking model, the hyper-parameters of the base learners and the meta learner were tuned with the last 20% of the original training dataset and the last 20% of the new training dataset, respectively. The grid search was applied, and the best hyper-parameter combinations were illustrated in Additional file 1: Table S2.

### Model evaluation

To demonstrate the superiority of our stacking model, we compared the stacking model with all base learners and LSTM [20] on the testing set. In the LSTM, the input variables only included historical HAs and environmental features, and the time window of the input sequence was set to 6, which was consistent with the lag days of other methods. The hyper-parameters of the benchmarks were also tuned with the last 20% of the original training dataset (shown as Additional file 1: Table S2).

Four metrics, including mean absolute error (MAE), root mean square error (RMSE), mean absolute percentage error (MAPE), and coefficient of determination (R^2^) were used to evaluate the effectiveness of the prediction models as Hu et al. [22].

### Model explainability

To explain the predictions of our final model, we made use of the permutation explainer implemented in the SHAP Python library (version 0.39.0). SHAP [40] is a unified approach based on the additive feature attribution method that interprets the difference between an actual prediction and the baseline as the sum of the attribution values, i.e., SHAP values, of each feature. In this study, the SHAP value for each feature in a given sample of CD dataset was calculated based on our proposed stacking model to present its contribution to the variation of HAs predictions. For the historical HAs and environmental features, their SHAP values were regarded as the sum of the SHAP values of all single-day lag and cumulative lag features, rendering their contributions during the previous 6 days.

A post-hoc interpretation was provided by analyzing the SHAP values from two perspectives. On the global scale, the SHAP values over all training samples were holistically analyzed to reveal how the stacking model fits the relationship between daily HAs and predictors. On the local scale, the SHAP values in several samples selected from the testing set were investigated to disclose how the predictions were generated in the effect of environmental features.

## Results

### Descriptive statistics

The summary statistics of daily HAs, air pollutants, and meteorological indicators are shown in Table 2, and the corresponding temporal variations of them are visualized in Additional file 1: Figs. S2 and S3, respectively. The correlations between environmental exposure variables are shown in Additional file 1: Table S3.

**Table 2:** Descriptive statistics of daily HAs for CD and environmental exposure data in Chengdu, 2015–2018

	Variables	Units	Mean	Std^a^	Min	25%	50%	75%	Max
Daily HAs	CD HAs	persons	70	35	6	42	68	96	214
	Stroke HAs	persons	45	21	4	27	43	60	131
Air pollutants	PM_2.5_	μg/m^3^	57.9	40.6	6.1	29.6	46.3	74.5	324.5
	PM_10_	μg/m^3^	95.7	62.1	12.0	51.6	78.4	124.5	492.5
	PM_C_	μg/m^3^	37.8	25.6	3.9	20.3	30.9	48.0	238.2
	O_3_	μg/m^3^	96.6	54.6	5.6	54.2	86.2	135.2	290.4
	SO_2_	μg/m^3^	12.7	5.5	3.9	8.5	11.2	15.3	37.9
	NO_2_	μg/m^3^	53.9	17.7	13.9	41.0	51.9	64.6	130.4
	CO	mg/m^3^	1.0	0.4	0.4	0.8	1.0	1.2	2.8
	AQI	1	85.2	48.5	16.7	52.5	71.4	103.8	404.6
Meteorological measures	TEM	℃	16.9	7.3	-1.1	10.1	17.4	23.2	30.2
	RH	%	80.5	9.2	43.0	74.4	80.8	87.7	99.3

^a^*Std* standard deviation

From 2015 to 2018, the total number of HAs for CD was 102,708, and the average number of daily HAs was 70 (std = 35). The daily mean ± std concentrations of PM_2.5_, PM_10_, PM_C_, O_3_, SO_2_, NO_2_, and CO were 57.9 ± 40.6, 95.7 ± 62.1, 37.8 ± 25.6, 96.6 ± 54.6, 12.7 ± 5.5, 53.9 ± 17.7, and 1030 ± 360 µg/m^3^, respectively. The value of AQI ranged from 16.7 to 404.6, with a mean of 85.2. The mean TEM and RH were 16.9℃ and 80.5%, respectively.

### Model performance

Table 3 compares the performance of base learners, LSTM, and the proposed stacking model on CD dataset and stroke dataset.

**Table 3:** Performance comparison of different methods in predicting HAs for CD and stroke

Datasets	Models	MAE	RMSE	MAPE	R^2^
**CD**	RF	14.713	20.649	0.154	0.652
	GBDT	14.661	20.296	0.154	0.663
	Ridge	14.894	19.963	0.183	0.674
	ANN	14.408	18.407	0.191	0.723
	LSTM	13.774	18.421	0.165	0.739
	Stacking	12.467	17.053	0.153	0.762
	Stacking + LDS	11.855^*^	16.078^*^	**0.145**	**0.789**
**Stroke**	RF	10.889	14.660	0.175	0.51
	GBDT	11.251	14.995	0.178	0.487
	Ridge	10.357	13.278	0.210	0.598
	ANN	9.525	12.140	0.191	0.664
	LSTM	9.422	12.166	0.185	0.676
	Stacking	9.038	11.898	0.170	0.677
	Stacking + LDS	8.961^*^	**11.850**	0.159^*^	**0.680**

The best result for each metric is in bold.

^*^The differences in the MAE, RMSE, or MAPE between the stacking model with LDS and the best individual model are significant (*P*-value < 0.05) according to the t-test

**Fig. 3:**
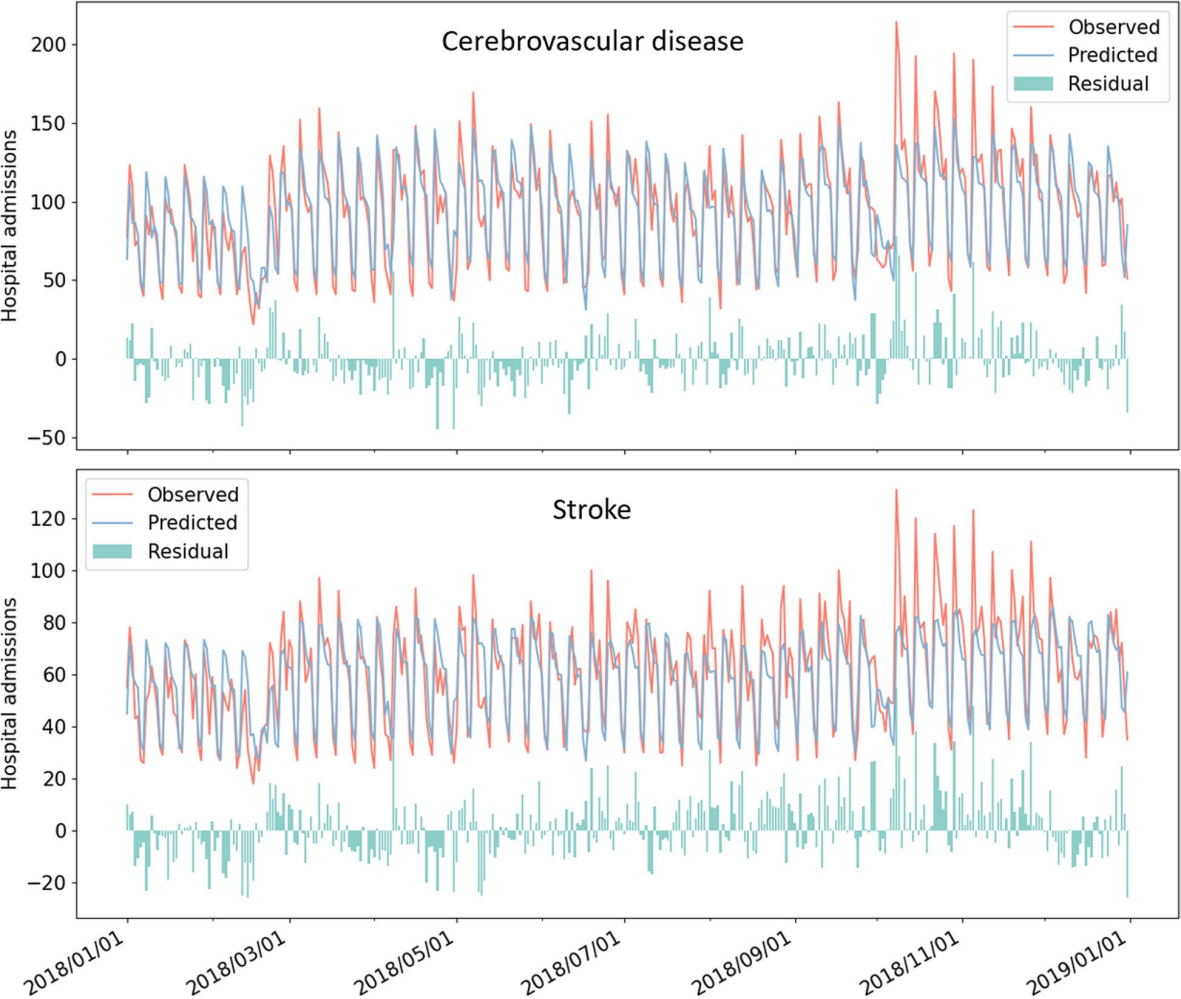
The comparison and residual between the observed HAs and the predictions of the stacking model with LDS on CD dataset and stroke dataset

On both datasets, ANN and LSTM surpass other individual models in terms of MAE, RMSE, and *R*^2^, but gain higher MAPE than tree-based models, and the stacking model is substantially superior to all individual models. After using LDS, the performance of the stacking model is further improved. On CD dataset, compared with the optimal results obtained by individual models, the MAE, RMSE, and MAPE of the stacking model with LDS remarkably reduced by 13.9%, 12.7%, and 5.8%, respectively, and the R^2^ increased by 6.8%. Additionally, the results of the t-test indicate that, when evaluated by most metrics, the performance gap between the stacking model with LDS and the best individual model is significant, and the difference between the R^2^ of them is visualized in Additional file 1: Fig. S4. Figure 3 shows a comparison between the observed HAs and the predictions of the stacking model with LDS on two datasets.

### Model explanation

#### Global explanation

**Fig. 4:**
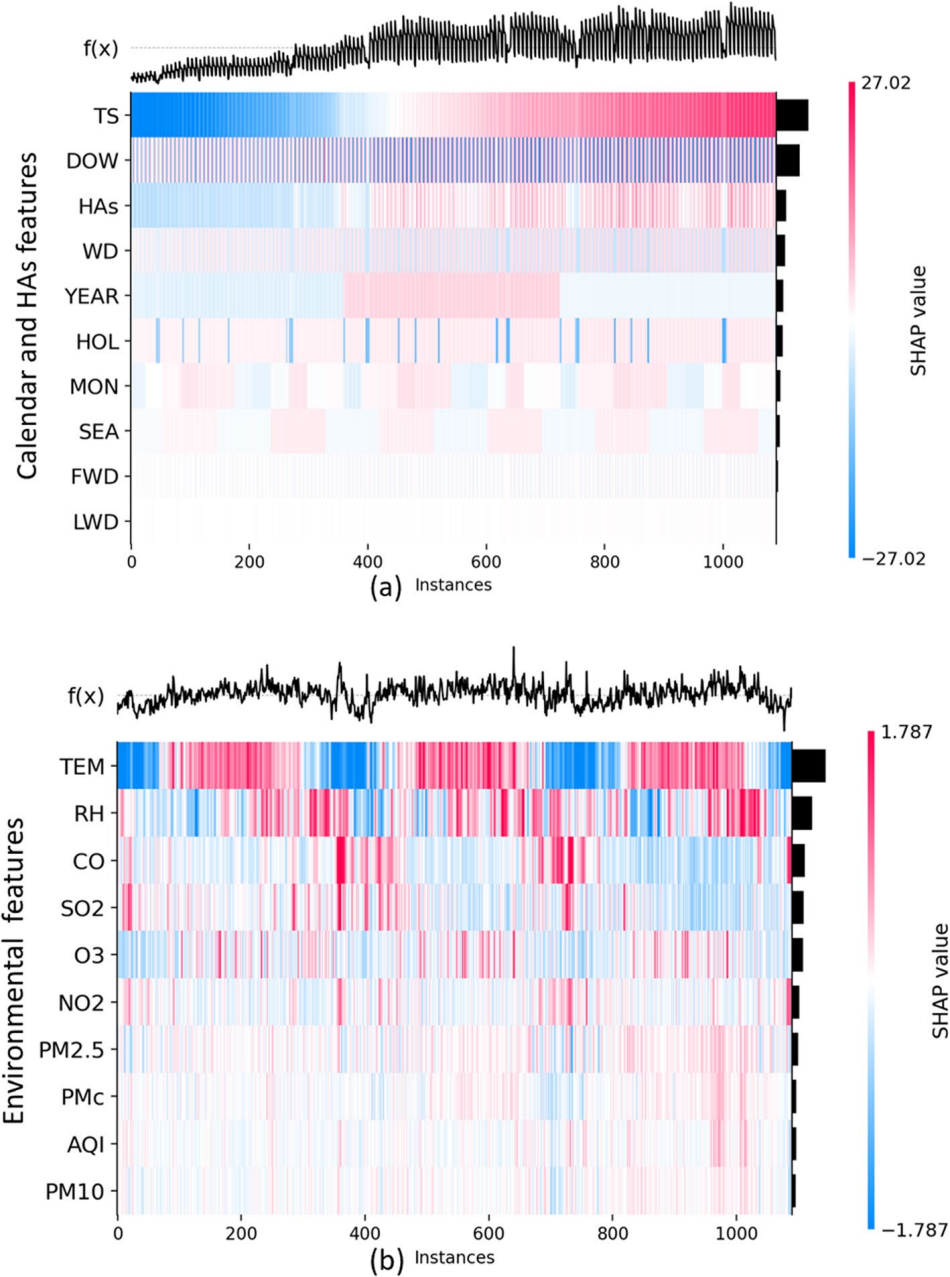
Heatmap plot of SHAP values of all features across all samples in the CD training set. The width of the black bar on the right-hand side shows the global importance of each feature. **a** Calendar features and HAs features **b** Environmental features

Figure 4 shows the distribution of SHAP values of each feature in chronological order, and the features are ranked according to the average of their absolute SHAP values over all the training samples, which represents their global importance.

Most straightforwardly, a calendar feature nearly always had similar SHAP values when it remained at the same values, resulting in visually prominent color blocks with a periodic alternation. In contrast, the impact of environmental features varied over samples without explicit patterns. In light of additive feature attribution theory, the predicted HAs could be regarded as the additive combination of three parts: a baseline (generally the average of predictions), a regular difference attributed by calendar features and historical HAs, and a highly volatile difference attributed by environmental features. As shown in Table 4, the second part depicted the general trend of variations in the number of daily HAs, and the third part served to further enhance the model performance.

**Table 4:** The improvement of model performance attributed to environmental features estimated by SHAP

	MAE	RMSE	MAPE	R^2^
Baseline + SHAP values of HAs features and calendar features	12.306	16.584	0.152	0.775
Final prediction	11.855	16.078	0.145	0.789
Improvement	3.665%	3.054%	4.306%	1.743%

After further observing each feature shown in Fig. 4, we found that TS and historical HAs played a major part in profiling the growth trend in HAs, and DOW served to depict the periodic variation of HAs caused by the work-rest schedule in hospitals (see Additional file 1: Fig. S2). TEM in the fall and summer contributed to increasing the predicted HAs. Notably, the annual peak concentrations of several air pollutants, such as O3, CO, and PM_2.5_, and their SHAP values pushing up the predicted HAs occurred at similar times (see Additional file 1: Fig. S3).

#### Local explanation

**Fig. 5:**
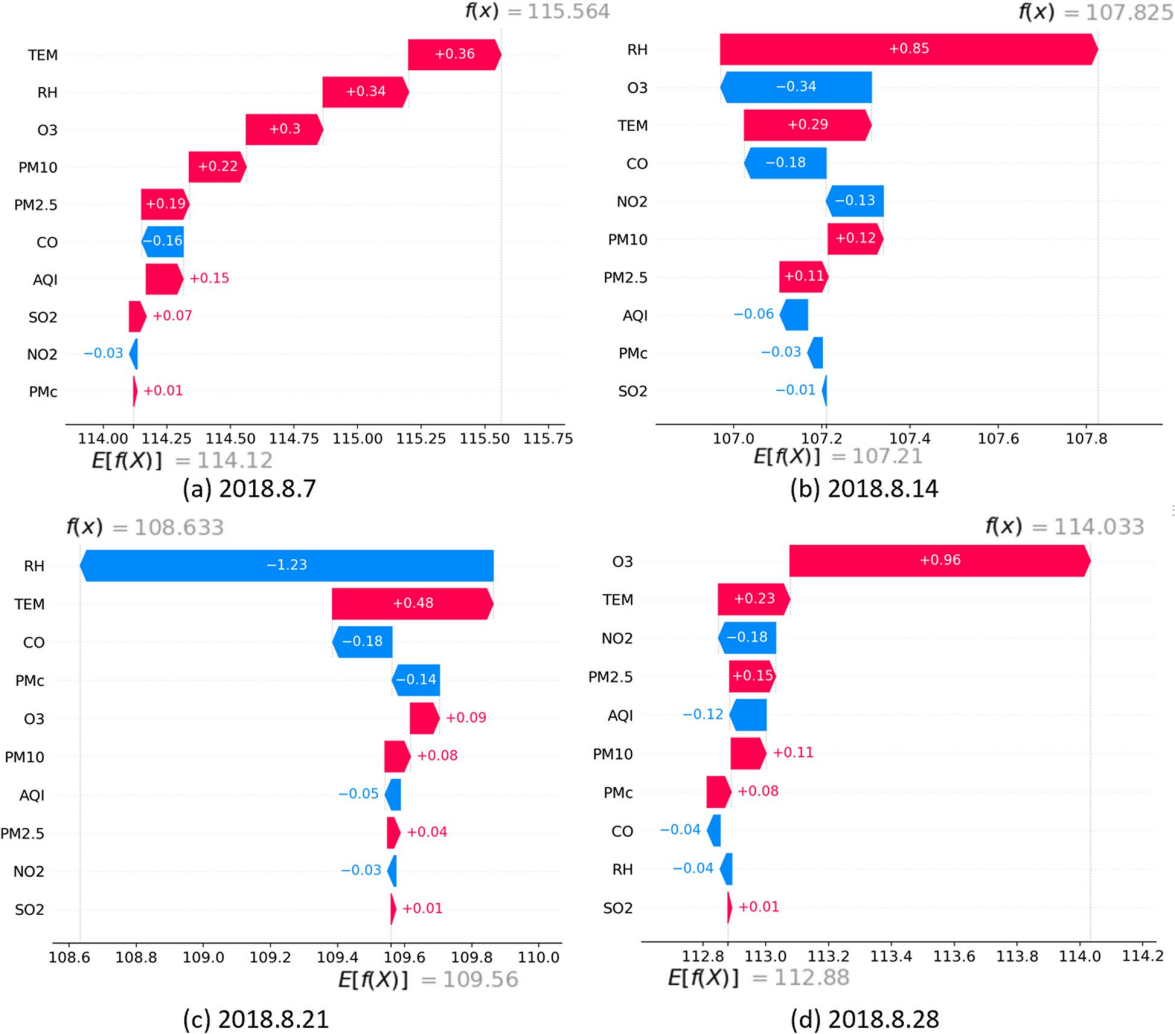
Waterfall plot of SHAP values to four selected samples, i.e., samples on August 7, 14, 21 and 28, 2018. The new baselines and the final predictions are marked at the bottom and top of the image, respectively. The SHAP values of each feature are listed on the bar

As the contributions of calendar features and HAs features are relatively straightforward and regular, it makes more sense to concentrate on how the involvement of environmental features improves the model performance. Thus, the samples on every Wednesday in August 2018 were selected to fix the calendar features except TS, where August was selected to further explore the impact of high temperatures on the risk of CD, and Wednesday was selected to reduce the interference caused by weekend breaks and Chinese holidays. The sum of the first two parts of the predictions mentioned above was set as a new baseline. Figure 5 shows how the SHAP values of environmental features were accumulated from the new baseline to reach the final predictions.

By comparing Fig. 5 and Additional file 1: Fig. S5, it was observed that the TEM in August and peak concentrations of PM_2.5_ and O_3_ that appeared in the six days leading up to August 28 served to increase the predicted values of HAs, while gradually declining RH over the previous 6 days lowered the predicted values on August 21 by around 1 count.

## Discussion

### Model performance analysis

**Fig. 6:**
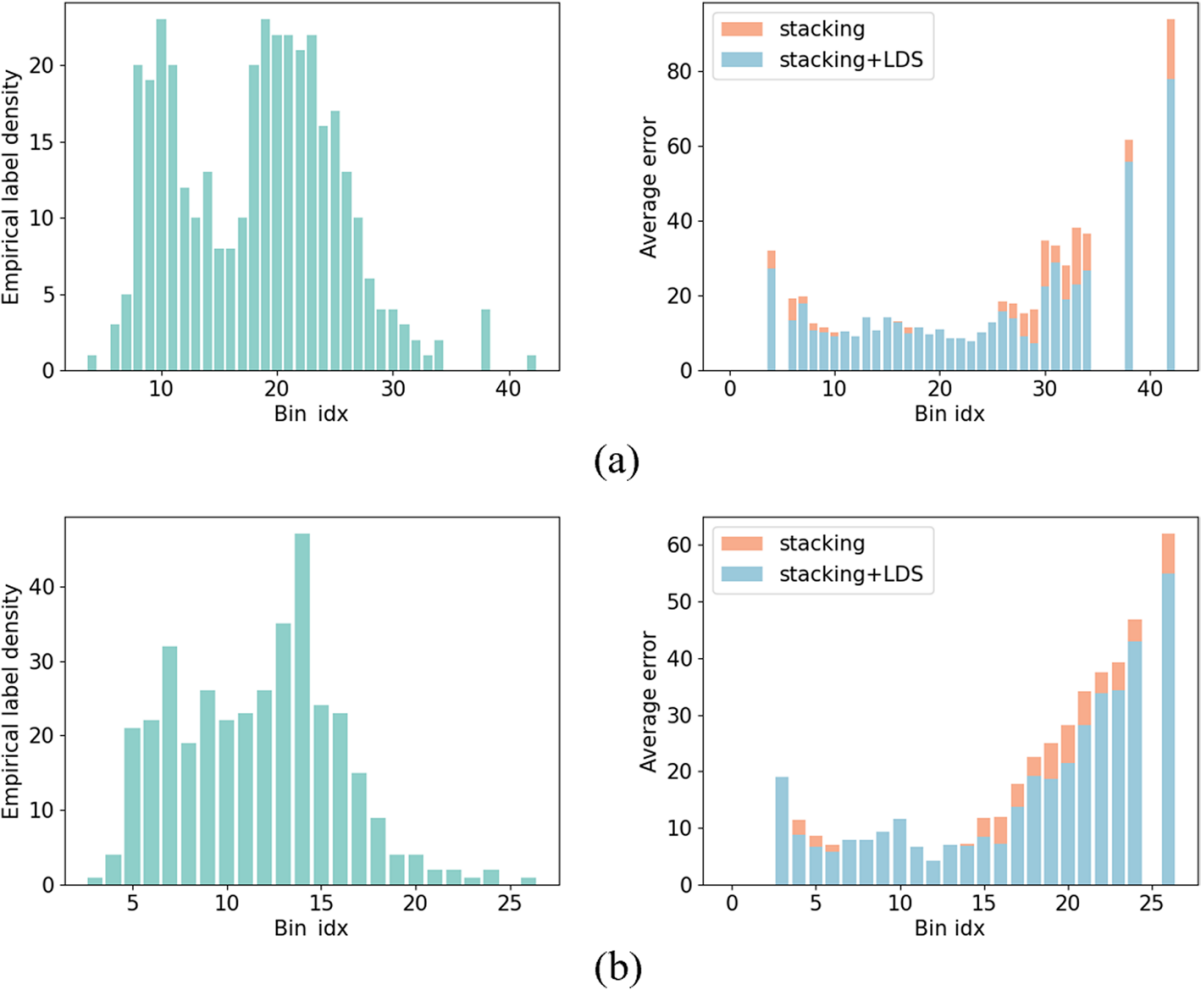
The left side shows empirical label distribution plots, and the right side shows comparison plots of error before and after using LDS on two testing datasets: **a** CD and **b** stroke

A distinct improvement of the stacking model compared to the base learners can be attributed to three aspects: 1) ANN and tree-based models performed best when evaluated by different metrics, which reflected the heterogeneity of their predictions and laid the foundation for meta learner to combine their strengths and obtain a better generalization [36]. 2) The key features served to help the meta learner understand how to choose and combine the predictions of base learners under various circumstances. 3) The re-weighting method based on LDS reduced the error caused by label imbalance, as shown in Fig. 6.

As shown in Additional file 1: Table S3, there are significant correlations among environmental exposure variables, however, the performance of the stacking model was not impacted because the base learners and the meta learner we selected can effectively handle multicollinearity. In Ridge and elastic net, the L2 regularization term added to their loss function can help stabilize the estimates and reduce overfitting in the presence of collinearity [41].

LSTM has been successfully applied in many fields, but in our task, its prediction performance is still inferior to the proposed stacking model. There are two potential reasons: 1) The size of our dataset is limited, with a total of 1461 samples, therefore, using lightweight models with simple architectures and fewer parameters can help avoid overfitting. 2) In our task, the time lag was set to 6 days to consider the short-term effect of environmental exposure, which constrained the advantage of LSTM in learning the long-time dependencies. However, the calendar features we extracted, such as YEAR, SEA, and MON, can assist the stacking model in capturing long-term trends.

### Comparing SHAP explanation and conventional association analysis

In our study, the explanations obtained by SHAP displayed a strong agreement with the conclusion of association analysis using conventional analytical methods based on statistical models, such as GLMs and generalized additive models (GAMs). For example, the TEM in summer and the peak values of air pollutant concentrations always played a role in describing the increase in HAs. This is consistent with the previous studies, which found high levels of air pollution and high temperatures were associated with a high morbidity of stroke [4, 5, 42, 43], but we did not observe that cold weather served to drive up the predicted HAs, probably because the minimum TEM (-1.1℃) during our research period in Chengdu did not reach the extreme cold defined in related studies. This consistency indicates that our stacking model can accurately describe the relationship between daily HAs for CD and environmental factors, thus improving the model performance.

**Fig. 7:**
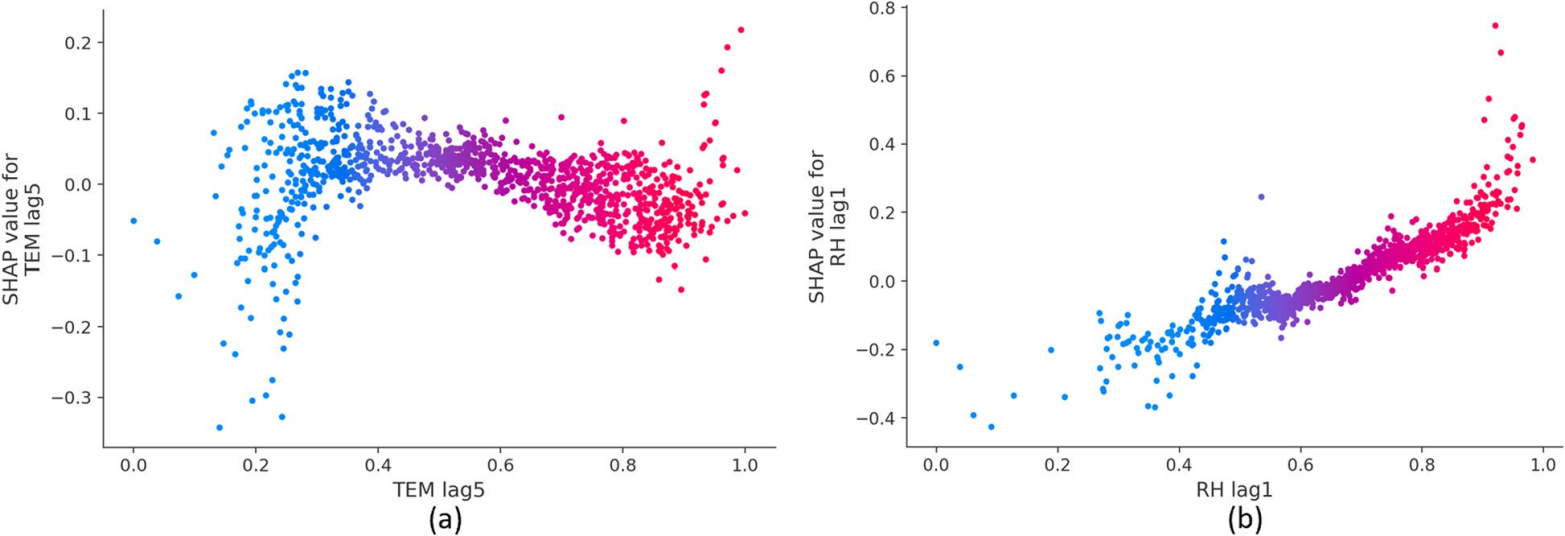
SHAP dependence plots that show the effect of TEM lag5 and RH lag1 on the predictions of HAs

Moreover, if we associate SHAP values that push up the predicted HAs with an increased risk of CD, the model explanations can be regarded as a new perspective to explore the adverse health effects of air pollutants and extreme weather conditions. For example, the relationship between the risk of CD and the lagged TEM and RH can be depicted in Fig. 7. Furthermore, SHAP can comprehensively account for the combined impact of multiple environmental factors, whereas most traditional methods can only analyze the association between single environmental factors and HAs after controlling confounding effects among multiple covariates by smoothing functions.

### Limitations

Several limitations should be addressed in our study. First, limited by the available data sources, we only considered the impact of ambient air pollutants and meteorological conditions on daily HAs for CD, but some other environmental factors and individual health behaviors may also play important roles in the development and severity of CD. Second, our proposed model is only applicable to predict HAs for non-communicable diseases, such as CD, which are associated with environmental exposure, and the model might not be suitable for forecasting the daily number of hospitalizations for infectious diseases. Third, the peak values of HAs are not well predicted, which could lead to under-allocation of medical resources.

## Conclusions

This study proposed a stacking ensemble model to predict the daily number of HAs for CD using the HAs data, air quality data, and meteorological data. The experimental results showed that our proposed model is superior to the base learners and LSTM on two datasets under four evaluation criteria. Moreover, the model explanation demonstrated that environmental factors played a role in further improving the model performance and identified that high TEM and high concentrations of gaseous air pollutant might strongly associate with an increased risk of CD. This study indicates that the proposed model considering environmental exposure factors is efficient in predicting daily HAs for CD and has practical value for hospital management teams in early warning and healthcare resource allocation.

## Supplementary Information

**Additional file 1: **An additional file provided supplementary figures and tables for comprehension of our research.

## Data Availability

The meteorological and air quality data are available at http://data.cma.cn/ and http://www.cnemc.cn/. Daily data of hospitalizations for cerebrovascular disease are available from the Health Information Center of Sichuan Province, but restrictions apply to the availability of these data, which were used under license for the current study, and so are not publicly available. The daily number of hospitalizations for cerebrovascular disease are however available from corresponding author on reasonable requests and with permission of the Health Information Center of Sichuan Province.

## Abbreviations

CD: Cerebrovascular disease
CVD: Cardiovascular disease
HAs: Hospital admissions
ML: Machine learning
SES: Simple exponential smoothing
ARIMA: Autoregressive integrated moving average
LSTM: Long Short-Term Memory
GLM: Generalized linear model
GAM: Generalized additive models
AQI: Air quality index
TEM: Temperature
RH: Relative humidity
DOW: Day of the week
MON: Month
SEA: Season
TS: Timestamp
HOL: Holiday
WD: Workday
FWD: First work day
LWD: Last work day
LDS: Label distribution smoothing
RF: Random forest
GBDT: Gradient boosting decision tree
ANN: Artificial neural network
MAE: Mean absolute error
RMSE: Root mean square error
MAPE: Mean absolute percentage error
R^2^: Coefficient of determination
SHAP: SHapley Additive exPlanations
