# Supplementary material for: Explainable prediction of daily hospitalizations for cerebrovascular disease using stacked ensemble learning
Source: BMC Med Inform Decis Mak. 2023 Apr 6;23:59. doi: 10.1186/s12911-023-02159-7 (PMC10080841; doi:10.1186/s12911-023-02159-7)
Supplement: Supplementary file 1 — Additional file 1: An additional file provided supplementary figures and tables for comprehension of our research. [file 12911_2023_2159_MOESM1_ESM.docx]

**Additional file 1**

Supplementary material for the article entitled “**Explainable prediction of daily hospitalizations for cerebrovascular disease using stacked ensemble learning**”.

**Table S1** The performances of eight candidates of base learners on the valid set.

| Model Types | Models | MAE | RMSE | MAPE | R^2^ |
| --- | --- | --- | --- | --- | --- |
| Tree | RF | 12.359 | 16.850 | 0.148 | 0.746 |
|  | GBDT | 12.465 | 16.551 | 0.149 | 0.755 |
|  | XGBoost | 14.809 | 19.978 | 0.165 | 0.643 |
|  | Adaboost | 17.901 | 21.706 | 0.234 | 0.579 |
| Linear | Lasso | 17.280 | 21.091 | 0.271 | 0.602 |
|  | Ridge | 14.351 | 19.097 | 0.193 | 0.674 |
|  | Elastic Net | 16.885 | 20.747 | 0.261 | 0.615 |
| Network | ANN | 14.802 | 19.043 | 0.211 | 0.676 |


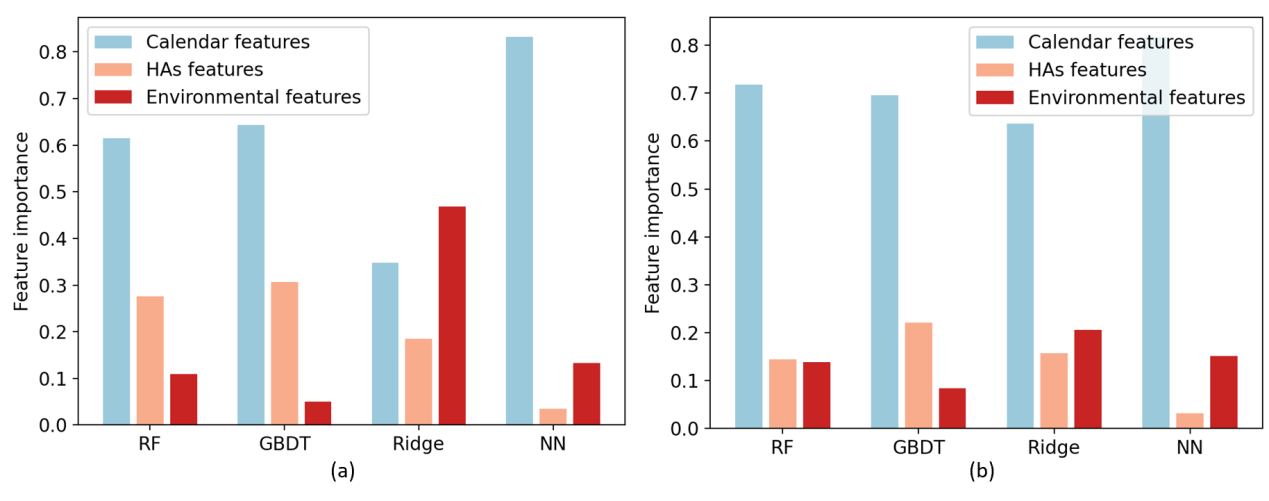


**Figure S1** The feature importance in base models on two datasets: (a) CD and (b) stroke. The feature importance in tree-based model, Ridge and ANN was calculated from impurity, regression coefficients and SHAP values, respectively. For each base learner and both datasets, historical HAs and calendar features accounted for less than 20% of the number of features, but more than 50% of the feature importance, therefore we defined them as the key features which would be reused by meta learner in the stacking model.

**Table S2** The range of grid search and the best hyper-parameter combinations in the stacking model and benchmarks.

| Model | Hyper-parameter name | The range of grid search | Best hyper-parameters (CD) | Best hyper-parameters (stroke) |
| --- | --- | --- | --- | --- |
| RF | max depth | [10, 20, 30, 40, None] | 20 | 30 |
|  | max features | [auto, sqrt, log2, None] | auto | auto |
|  | n estimators | [20, 30, 40, 50] | 40 | 40 |
| GBDT | learning rate | [0.05, 0.1, 0.15, 0.2] | 0.1 | 0.15 |
|  | max depth | [3, 6, 9, 12] | 3 | 3 |
|  | max features | [auto, sqrt, log2, None] | None | auto |
|  | n estimators | [80, 90, 100, 110] | 100 | 100 |
| Ridge | alpha | [0.90, 0.91,0.92, … , 1.10] | 1.09 | 1.09 |
| ANN | Number of layers | Increase until the performance in the valid set is no longer optimized | 2 | 2 |
|  | Units of the first layer | [16, 32, 48, 64, 80] | 48 | 32 |
|  | Units of the second layer | [8, 16, 24, 32] | 16 | 16 |
|  | Activation function | [Tanh, ReLU, Sigmoid] | ReLU | ReLU |
|  | Learning rate | [0.0005, 0.001, 0.005, 0.01] | 0.001 | 0.001 |
|  | Loss function | [MSE, MAE] | MSE | MSE |
|  | Optimizer | [Adam, SGD, RMSprop] | Adam | Adam |
|  | Batch size | [8, 16, 32, 64, 128] | 64 | 32 |
|  | Training epochs | [250, 500, 1000, 1500] | 1000 | 500 |
| Elastic Net | alpha | [0.01, 0.02,0.03, … , 0.10] | 0.02 | 0.03 |
|  | l1 ratio | [0.1, 0.2, … , 1] | 0.9 | 0.9 |
| LSTM | Number of hidden layers | Increase until performance in the valid set is no longer optimized | 1 | 1 |
|  | Units of the hidden layer | [16, 32, 64, 128] | 64 | 64 |
|  | Number of fully connected layers | Increase until the performance in the valid set is no longer optimized | 1 | 1 |
|  | Learning rate | [0.0005, 0.001, 0.005, 0.01] | 0.001 | 0.001 |
|  | Loss function | [MSE, MAE] | MSE | MSE |
|  | Optimizer | [Adam, SGD, RMSprop] | Adam | Adam |
|  | Batch size | [8, 16, 32, 64, 128] | 32 | 16 |
|  | Training epochs | [100, 250, 500, 1000] | 500 | 250 |

* Hyper-parameters not listed in the table are default values in scikit-learn 0.24.2 and Tensorflow 2.4.1.


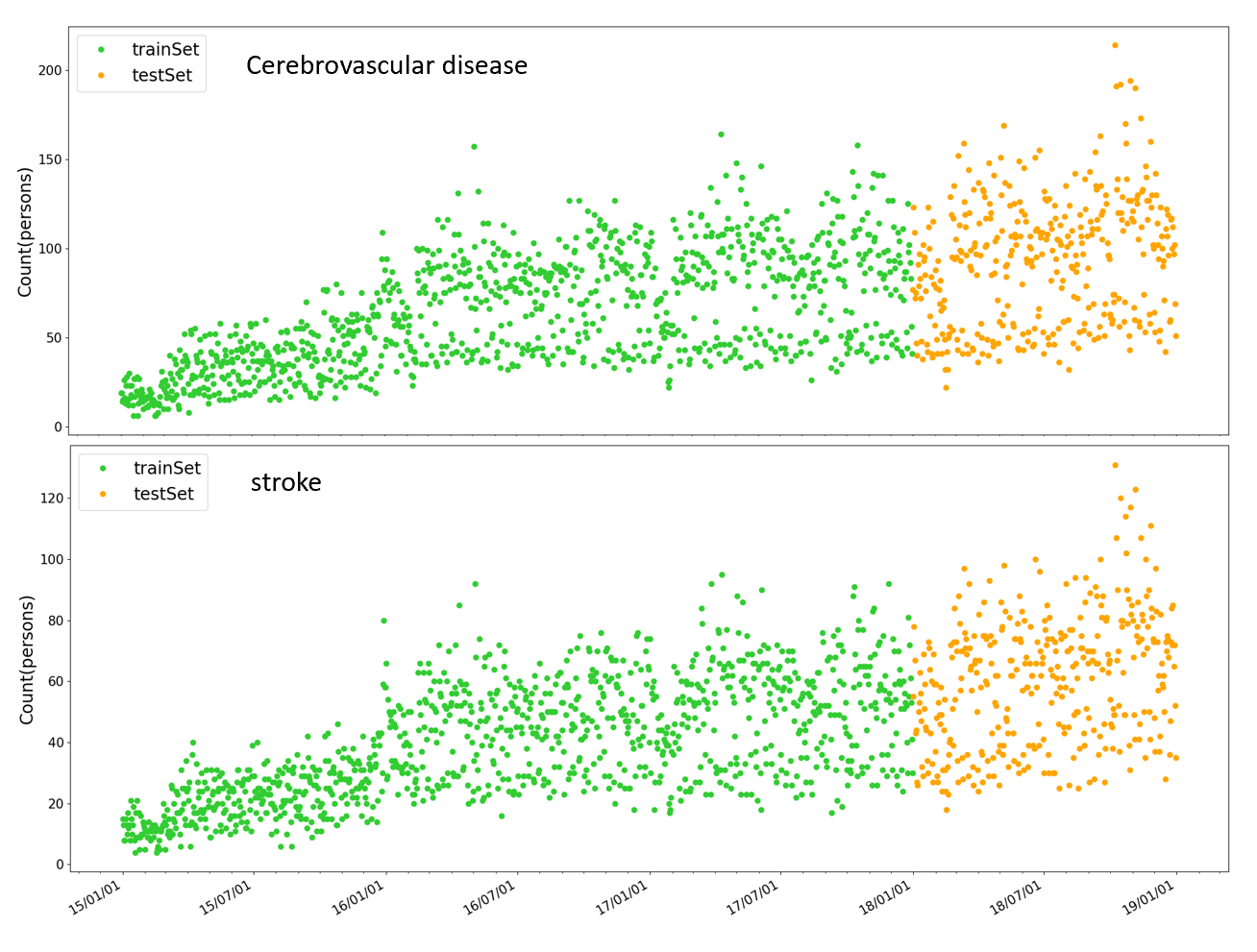


**Figure S2** Temporal variations of HAs for CD and stroke during 2015/1/1 through 2018/12/31 in Chengdu, China.

**Table S3** Pearson correlations between environmental exposure variables.

|  | AQI | PM_10_ | PM_c_ | PM_2.5_ | SO_2_ | NO_2_ | CO | O_3_ | TEM | RH |
| --- | --- | --- | --- | --- | --- | --- | --- | --- | --- | --- |
| AQI | 1.00 |  |  |  |  |  |  |  |  |  |
| PM_10_ | 0.98^*^ | 1.00 |  |  |  |  |  |  |  |  |
| PM_c_ | 0.81^*^ | 0.90^*^ | 1.00 |  |  |  |  |  |  |  |
| PM_2.5_ | 0.99^*^ | 0.96^*^ | 0.75^*^ | 1.00 |  |  |  |  |  |  |
| SO_2_ | 0.59^*^ | 0.61^*^ | 0.56^*^ | 0.59^*^ | 1.00 |  |  |  |  |  |
| NO_2_ | 0.75^*^ | 0.77^*^ | 0.69^*^ | 0.74^*^ | 0.62 ^*^ | 1.00 |  |  |  |  |
| CO | 0.80^*^ | 0.80^*^ | 0.63^*^ | 0.82^*^ | 0.64^*^ | 0.72^*^ | 1.00 |  |  |  |
| O_3_ | -0.20^*^ | -0.22^*^ | -0.11^*^ | -0.27^*^ | -0.04 | -0.07^*^ | -0.27^*^ | 1.00 |  |  |
| TEM | -0.45^*^ | -0.45^*^ | -0.32^*^ | -0.48^*^ | -0.15^*^ | -0.28^*^ | -0.41^*^ | 0.71^*^ | 1.00 |  |
| RH | -0.07^*^ | -0.09^*^ | -0.20^*^ | 0.00 | -0.15^*^ | -0.12^*^ | 0.07^*^ | -0.46^*^ | -0.03 | 1.00 |

Note: *two-tailed test of significance is used (*P*-value<0.05).


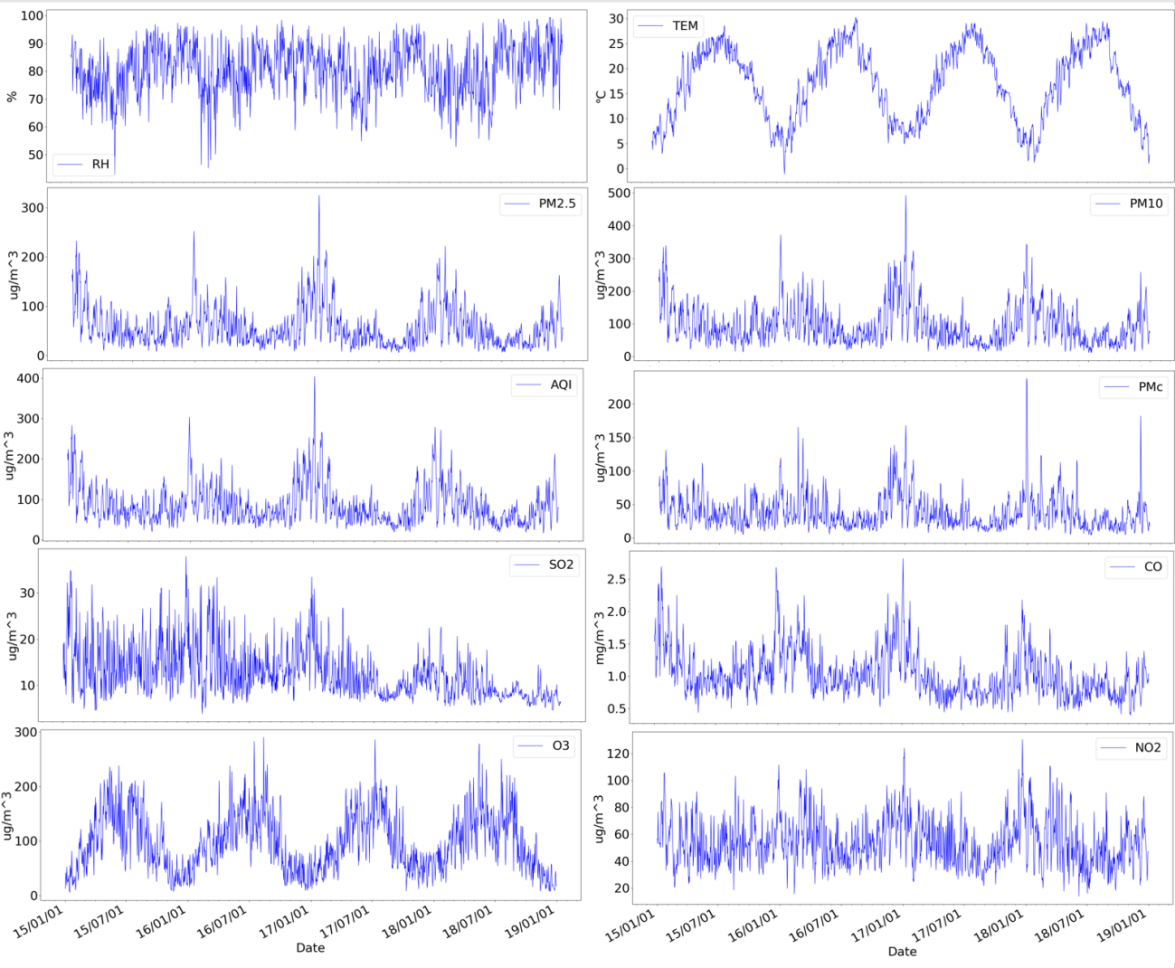


**Figure S3** Temporal variations of environmental exposure data during 2015/1/1 through 2018/12/31 in Chengdu, China.


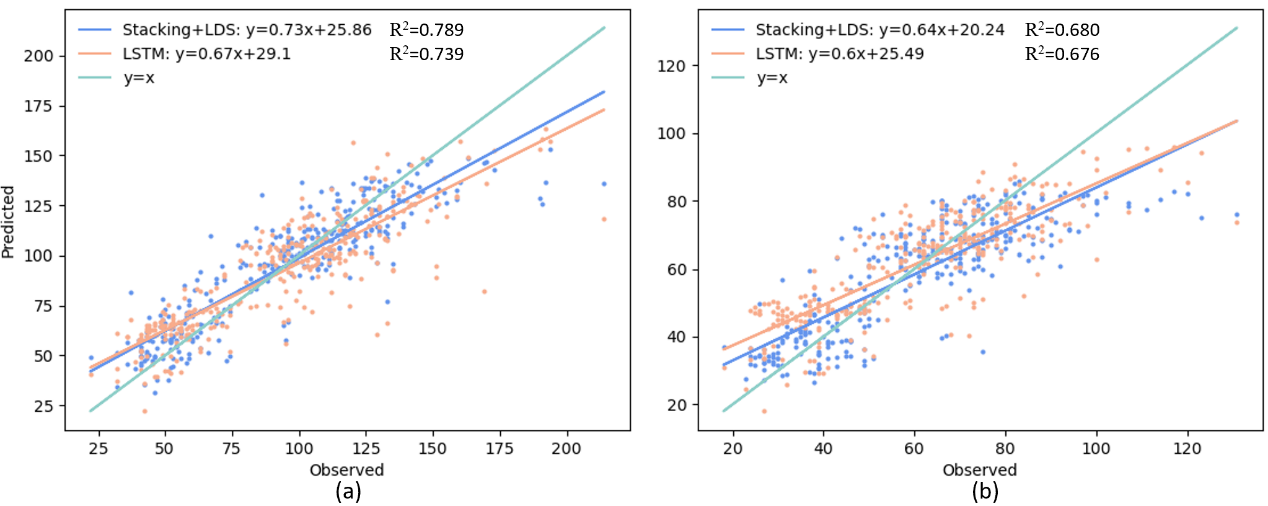


**Figure S4** The scatter plots to compare the R^2^ of the stacking model and the R^2^ of the best individual model (LSTM) on two datasets: (a) CD and (b) stroke.


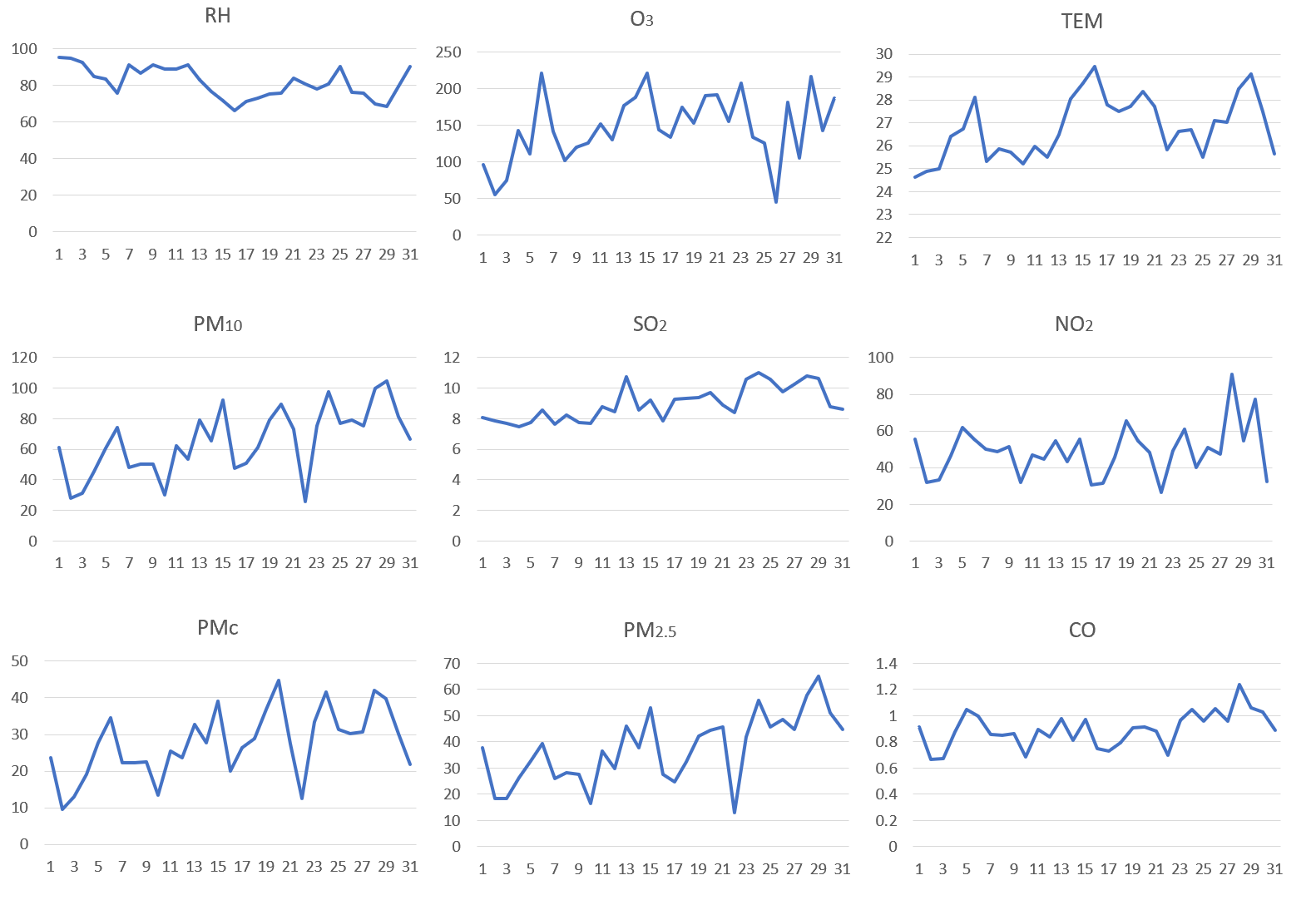


**Figure S5** Temporal variations of environmental factors during August 2018. The units of TEM, RH and CO are ℃, % and mg/m^3^, respectively. The units of other factors are µg/m^3^.
